# Sedimentology and ichnology of the Mafube dinosaur track site (Lower Jurassic, eastern Free State, South Africa): a report on footprint preservation and palaeoenvironment

**DOI:** 10.7717/peerj.2285

**Published:** 2016-08-23

**Authors:** Lara Sciscio, Emese M. Bordy, Mhairi Reid, Miengah Abrahams

**Affiliations:** Department of Geological Sciences, University of Cape Town, Cape Town, South Africa

**Keywords:** Karoo, Elliot formation, Casting, Theropoda, Dinosaur tracks, Lower Jurassic, Early Jurassic

## Abstract

Footprint morphology (e.g., outline shape, depth of impression) is one of the key diagnostic features used in the interpretation of ancient vertebrate tracks. Over 80 tridactyl tracks, confined to the same bedding surface in the Lower Jurassic Elliot Formation at Mafube (eastern Free State, South Africa), show large shape variability over the length of the study site. These morphological differences are considered here to be mainly due to variations in the substrate rheology as opposed to differences in the trackmaker’s foot anatomy, foot kinematics or recent weathering of the bedding surface. The sedimentary structures (e.g., desiccation cracks, ripple marks) preserved in association with and within some of the Mafube tracks suggest that the imprints were produced essentially contemporaneous and are true dinosaur tracks rather than undertracks or erosional remnants. They are therefore valuable not only for the interpretation of the ancient environment (i.e., seasonally dry river channels) but also for taxonomic assessments as some of them closely resemble the original anatomy of the trackmaker’s foot. The tracks are grouped, based on size, into two morphotypes that can be identified as *Eubrontes*-like and *Grallator*-like ichnogenera. The Mafube morphotypes are tentatively attributable to large and small tridactyl theropod trackmakers, possibly to *Dracovenator* and *Coelophysis* based on the following criteria: (a) lack of manus impressions indicative of obligate bipeds; (b) long, slender-digits that are asymmetrical and taper; (c) often end in a claw impression or point; and (d) the tracks that are longer than broad. To enable high-resolution preservation, curation and subsequent remote studying of the morphological variations of and the secondary features in the tracks, low viscosity silicone rubber was used to generate casts of the Mafube tracks.

## Introduction

The fluvio-lacustrine and aeolian rocks of the Upper Triassic - Lower Jurassic Elliot and Clarens formations (Karoo Supergroup) preserve not only a range of vertebrate body fossils, but also a plethora of vertebrate invertebrate and plant ichnofossils that collectively record information about the dynamics of a ∼200 Ma old palaeoecological system in southern Africa (Ellenberger, 1970; Kitching & Raath, 1984; Bordy, Hancox & Rubidge, 2004). Furthermore, these fossiliferous rocks were deposited before and after the third largest of five major biological crises which occurred during the Phanerozoic. Characterising the ecological changes that occurred due to this major mass extinction event in southern Africa has the potential to inform hypotheses concerning the causes of this extinction as well as the adaptation strategies of the biota after the event.

**Figure 1:**
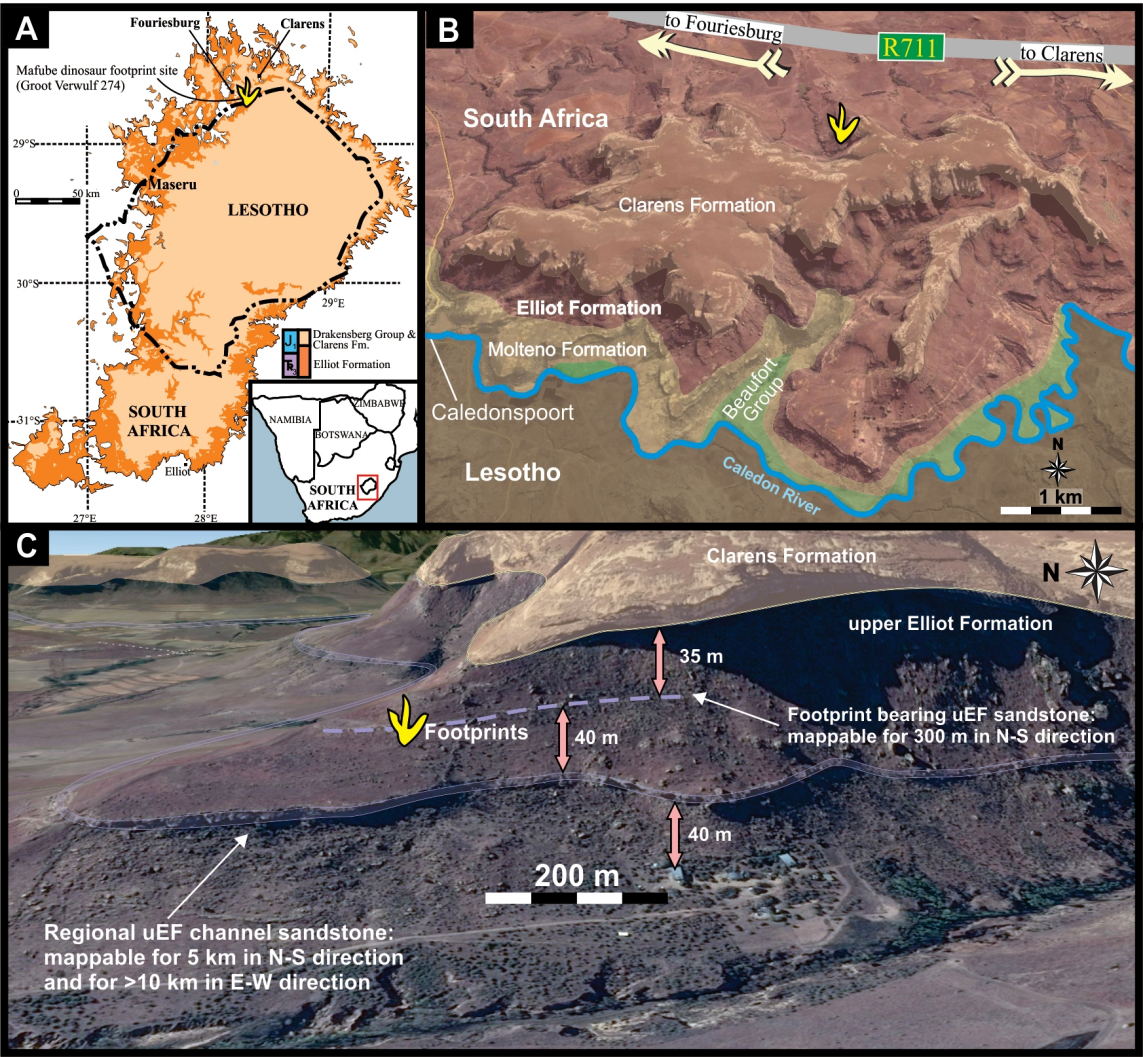
Location and stratigraphy of the Mafube dinosaur track site. (A) Geological map of the Elliot Formation in the Republic of South Africa and Lesotho (modified after the 1:1000000 Geological map of RSA and Lesotho, 1984). (B) Simplified regional geological map overlay onto Google Earth image of the vicinity of the Mafube dinosaur track site (Lower Jurassic, eastern Free State, South Africa). Map Data: AfriGIS (Pty Ltd.), Google, DigitalGlobe, CNES/Astrium. (C) Landscape view, taken from Google Earth, of the study site at Mafube showing aspects of the local geology. Mafube means “The dawning of a new day” in SeSotho. Map Data: AfriGIS (Pty Ltd.), Google, DigitalGlobe, CNES/Astrium.

The vertebrate footprints in the Upper Triassic to Lower Jurassic Elliot Formation have been assigned to multiple tetrapod genera some 45 years ago largely by Ellenberger (1970), Ellenberger (1972) and Ellenberger (1974), revised some 30 years later by Olsen & Galton (1984), and more recently reviewed again by  D’Orazi Porchetti & Nicosia (2007). These studies focused on morphological descriptions, and did not take into account the sedimentological or taphonomic contexts of the footprint sites with the exception of the Wilson, Marsicano & Smith (2009) study at one location in Lesotho. Our detailed study of the theropod tracks and associated sedimentary structures at Mafube Mountain Retreat (eastern Free State, South Africa; Fig. 1) aims to determine not only the conditions under which the tridactyl dinosaur tracks were preserved, but also to provide additional data concerning the dynamics of the ancient ecosystem in the Early Jurassic.

The Mafube tracks are preserved as impressions (negative relief or concave epireliefs) of the original foot on the upper bedding plane of a very finegrained, horizontally laminated sandstone that is 40 m below the contact between the Elliot and Clarens formations (Fig. 1). In addition to their palaeoenvironmental significance, the casts of desiccation cracks found in some of the Mafube tracks add to the importance of the site for ichnotaxonomic interpretations. They indicate that the tracks are true tridactyl dinosaur tracks rather than undertracks, and therefore the track dimensions accurately reflect for the trackmaker’s original foot anatomy.

The Mafube tracks also highlight the importance of refining the ichnotaxonomy of Lower Jurassic tridactyl tracks known for their superficially similar track morphology. The results help convey the relevance of the debate surrounding the *Grallator-Anchisauripus-Eubrontes* plexus (Olsen, 1980) and the value (Rainforth, 2005) or lack thereof (Lucas et al., 2006) for synonymising ichnogenera that can only be reliably distinguished by size (Lucas et al., 2006; Lockley, 2010).

### Geological background and stratigraphy of the tracksite

The Mafube dinosaur track site is located between Fouriesburg and Clarens in the eastern Free State, South Africa (Fig. 1) on the properties of Mafube Mountain Retreat (formerly known as Groot Verwulf 274, and prior to that as eastern Blackwood 329). The region is dominated by the Upper Triassic to Lower Jurassic clastic sedimentary rocks of the Stromberg Group (Molteno, Elliot and Clarens formations) as well as the 183 ± 1.0 Ma old intrusive and extrusive mafic rocks of the Drakensberg Group (Duncan et al., 1997; Fig. 1).

Stratigraphically, the track site is ∼35 m below the base of the Clarens Formation, and within the last of last sandstone of the Elliot Formation (Fig. 1C). This Upper Triassic-Lower Jurassic fluvio-lacustrine unit has an unconformable, sharp, regionally traceable lower contact with the fluvio-lacustrine and coal-bearing Molteno Formation and a conformable, chiefly gradational upper contact with the mainly aeolian Clarens Formation (Bordy, Hancox & Rubidge, 2004). The Elliot Formation crops out as a ring-shaped belt surrounding the Drakensberg Plateau and has a maximum thickness of nearly 500 m in the south that thins to <30 m in the north (Botha, 1967; Smith & Kitching, 1997). Near the study site, the unit ranges in thickness from ∼160–185 m in the region south of Mafube, and in northern Lesotho to about ∼120 m just east of Fouriesburg. Within the Elliot Formation there are major sedimentary facies differences which allow the subdivision of the formation into two informal units, namely the lower and upper Elliot formations (lEF and uEF; Bordy, Hancox & Rubidge, 2004).

Regionally, the upper Elliot formation consists of silty mudstones with intermittent sandstones that have a distinctive deep red to maroon colour with erratic, light grey mottles and an abundance of other pedogenic features (Bordy, Hancox & Rubidge, 2004). The sandstones of uEF are sheet-like, tabular and multi-storied bodies that are tens of metres wide and up to 6 m thick. Individual beds within the sandstone bodies have thicknesses ranging between 0.2–1 m and are separated by flat, internal erosional surfaces with geometries similar to the basal bounding surface of the multi-storied sandstone bodies. The bounding surfaces in the uEF sandstones are laterally continuous, parallel, and devoid of topographical irregularities greater than tens of centimetres (Bordy, Hancox & Rubidge, 2004). Internally, the tabular fine to very finegrained sandstones of the uEF are dominated by massive beds, horizontal lamination, low-angle cross-bedding, parting lineations, ripple cross-lamination, flaser and wavy bedding, mud draped surfaces, small-scale soft sediment deformations and bioturbation (Bordy, Hancox & Rubidge, 2004). Channel lags, comprising pedogenic nodule conglomerates, commonly occur at the base of sandstone bodies. These nodules range from rounded to sub-angular, are moderately sorted and white to red in colour. In addition to the pedogenic glaebule conglomerates (a diagnostic lithology of the uEF) mud pebble conglomerates may also form stingers at the base the upward fining successions.

The uEF sandstones are separated from one another by 0.5–10 m thick mudstone units that range from pure claystone to fine-sandy siltstone which are mostly massive but sometimes horizontally laminated (Bordy, Hancox & Rubidge, 2004). The mudstones contain *in situ* pedogenic calcareous concretions, irregular mottles, desiccation cracks, falling water level marks and mud drapes (Bordy, Hancox & Rubidge, 2004). The mudstones also contain a large diversity of vertebrate body fossils, including sauropodomorph dinosaurs, turtles, fishes, amphibians, crocodylians, advanced therapsids and early mammals as well as crustaceans (conchostracans) petrified wood, and carbonised and calcretised root traces (Bordy & Eriksson, 2015). The informal lithostratigraphic units of the Elliot Formation coincide with the informal biostratigraphic units of Kitching & Raath (1984), namely the ‘*Euskelosaurus*’ and *Massospondylus* Range Zones with the lEF and uEF, respectively. The Mafube dinosaur track site therefore falls within the *Massospondylus* Range Zone, and this is supported by the fossil remains reported from Mafube, which include the postcranial remains (scapula, coracoid and tarsal) of *Massospondylus* sp together with crocodylians remains (*Protosuchus haughtoni*) and a jaw from a large, basal archosaurian reptile (‘thecodont’, ?rauisuchid; Kitching & Raath, 1984). Less than 9 km from the Mafube track site, the following fossil remains have been reported in Kitching & Raath (1984): (a) dinosaurs (mostly *Massospondylus* sp.) from farms Glen Skye (121), Helpmekaar (251), La France (379), Saaihoek (194), Bloemhoek (330), Naudes Lust (334), Mizpah (164), De Villiersdrift (338), Langkloof (34), and Foutanie (331); (b) cynodonts (only *Tritylodon* sp.) from farms Saaihoek (194), Bloemhoek (330), Naudes Lust (334), and Langkloof (34); (c) basal archosaurian reptile (‘thecodont’, ?rauisuchid) from farms Foutanie (331); and (d) amphibians (skull and jaw fragments) from farms De Villiersdrift (338) and Langkloof (34). It is noteworthy that a large partial femur of the “Highland Giant,” a herbivorous sauropod was discovered only <17 km ENE of Mafube at the same stratigraphic level as the Mafube track site in close proximity to the contact of the Elliot and Clarens formations (McPhee et al., 2016).

## Material and Methods

Field data were collected in the form of macroscopic observations of the ichnofossil bearing sedimentary rocks as well as by recording the vertical and lateral distributions of the sedimentary structures at the study locality. The outcrop was photographed and described with sufficient detail to produce an in depth characterization of the sedimentary facies. This entailed the documenting of the lithology, geometry, sedimentary structures, palaeocurrent and fossil occurrences at centimetre scale resolution. Wellpreserved tracks were also measured and recorded in detail via photographs, photomosaics and sketches. Field observations were graphically summarised in annotated photographs to enhance the data presentation and analysis.

**Figure 2:**
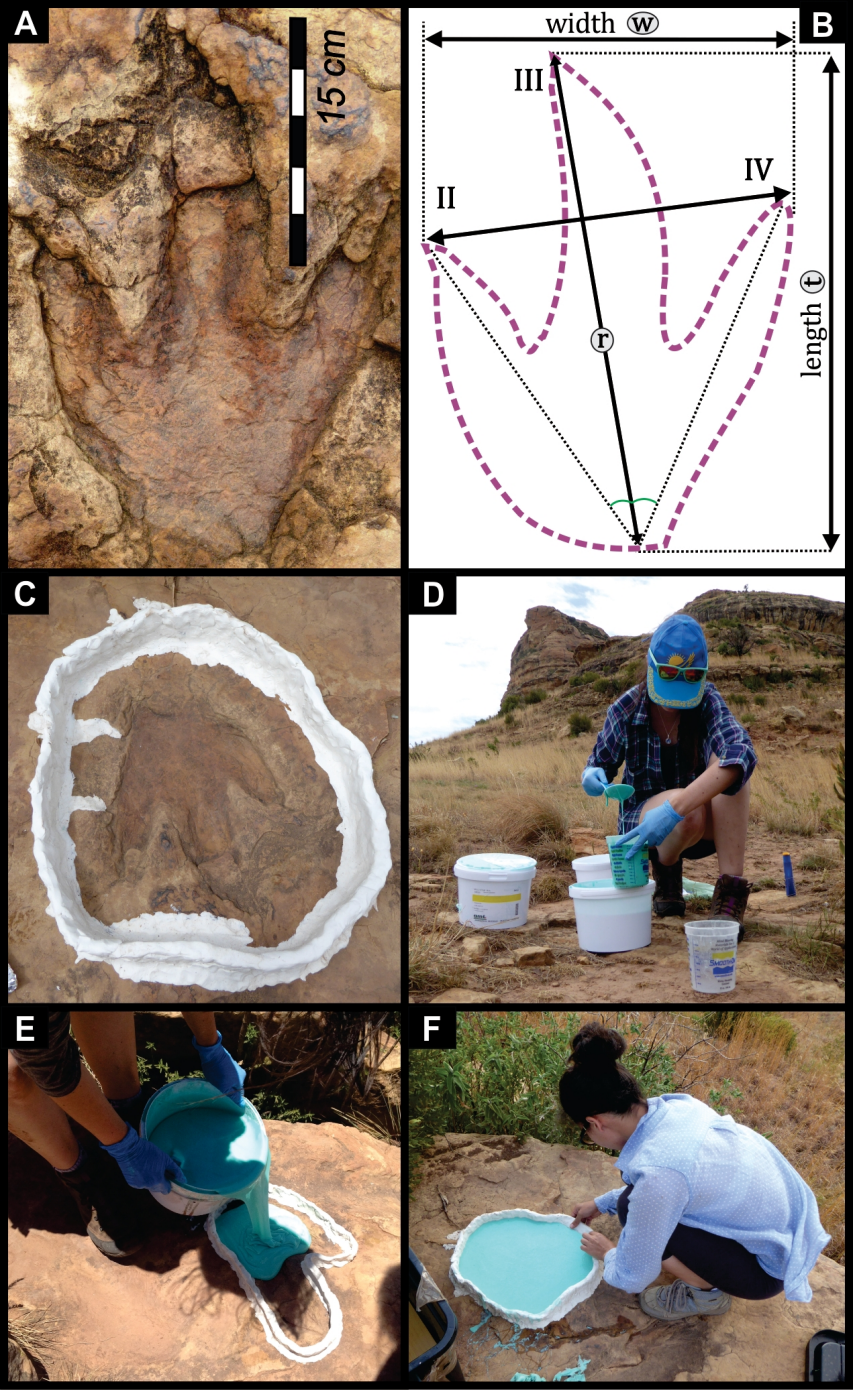
Track morphology (A) and corresponding outline drawing (B) of a Mafube track showing the placement of measurements used in this study. Numbers II–IV are referencing the imprints of the three digits of a tridactyl theropod dinosaur foot; interdigit angles (marked with green arches) are also referred to as divarication angles; ‘t’ is the track length; ‘w’ is the track width; ‘r’ is the length of rear of phalangeal part of foot (cf. Olsen, Smith & McDonald, 1998). Footprint casting process documented in photographs. (C) A cleaned and dried track has been surrounded with a retaining wall of non-curing modelling clay. (D) Decanting Part A and Part B of the silicone rubber into two measuring containers in equal amounts. (E) Pouring the silicone rubber into a track. (F) Removing the non-curing modelling clay to free-up the cured rubber cast of the track.

Track terminology and morphologically descriptive terms used here are based on vertebrate body fossil anatomy of tridactyl theropod dinosaurs (Figs. 2A and 2B; Table 1; Table S1). Digit numbering is a function of the three toes which make up the tridactyl foot of a theropod dinosaur with the middle digit (digit III) being the longest, and digits II and IV being subequal in length (Figs. 2A and 2B; Lockley, 2010). Digit I, referred to as the hallux, is generally not preserved in tracks, unless the sediment consistency allows for this otherwise anatomically elevated digit to be impressed into or enveloped by the substrate. The hypex is the connective area between the digits II and III and between III and IV. The interdigit divarication angle represents the angle between the digits II and III as well as III and IV. This is measured between the midline centred on digit III and a midline running through digit II and IV (Figs. 2A and 2B). Tracks were analysed using the following parameters: (1) track length (t); (2) track width (w); and (3) the ratio between the length and width (t/w) (Figs. 2A and 2B). Metatarsal impressions are rare imprints of the sole of the foot on the sediment. Measurements were obtained from the tracks in the field and later, from casts and line drawings of photographs.

Using a low viscosity silicone rubber, four casts of the over 80 tridactyl tracks preserved at Mafube were obtained. The most well-preserved tracks were chosen: track #3 (Surface I), track #31 (Surface II) and track #57 (Surface III). For casting of the tracks, we used a platinum cure, two-part component silicone rubber (Mold Star® 15 Smooth-On, Inc). The choice to use room temperature vulcanization (RTV) silicone rubber over latex rubber was due to the low levels of shrinkage, high strength and flexibility, as well as the long shelf life of the former. What makes the RTV silicone rubber ideal for field use is its pot life of 50 min, a short curing time (4 hours at 25°C), and a mixing ratio of one to one of its components (i.e., no need for measuring scales). Prior to casting, the four chosen tracks were carefully cleaned with a wet brush and then dried naturally. Individual tracks were surrounded by a retaining wall of non-curing modelling clay in order to prevent the silicone rubber from spilling beyond the foot impression (Fig. 2C). This, however, was not required for every track as some had naturally elevated margins that acted as containing walls. Equal amounts of the two-part component rubber, Part A and Part B were mixed carefully preventing the formation of air bubbles (Fig. 2D). This silicone rubber mixture was poured near the deepest point of the impression and to minimise the generation of air bubbles the rubber was then very slowly poured in a thin stream onto the inner side of the retaining wall or at the edge of the foot impression (Fig. 2E). After ∼45 min of curing at 35°C, the rubber was firm enough to be easily lifted from the dinosaur foot impression (Fig. 2F). Finally, the site was thoroughly cleaned and returned to its natural state. Silicone casts are housed in the Ichnology Collection of the Evolutionary Studies Institute at the University of Witwatersrand, South Africa.

## Results

### Description of the host rock sedimentology

**Figure 3:**
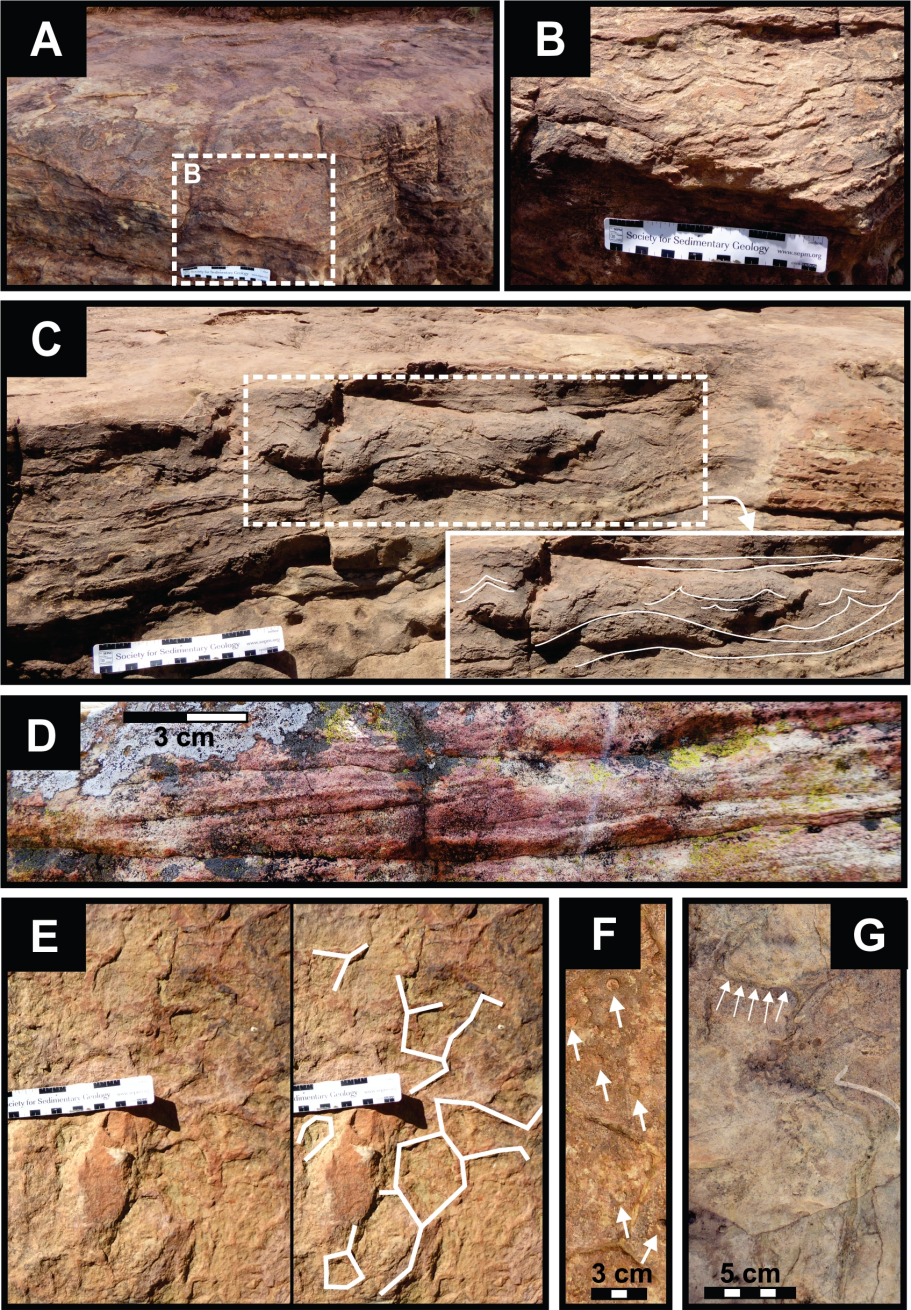
Sedimentary structures in the very fine grained sandstone host rock. (A) Close up of the horizontally laminated sandstone and its imprinted upper bedding surface (for position of B, see white rectangle). (B) Soft sediment deformation structures are common, especially close to the upper bedding plane. (C) Vertical section showing soft sediment deformation directly beneath depressions on the upper bedding plane of the horizontally laminated sandstoneare interpreted as deformation related to the loading effect by dinosaur foot. White rectangle marks the position of the inset with undertrack deformation (deformations marked in white). (D) Low-angle cross-bedding is present but rare. (E) Hexagonal casts of desiccation cracks on the exposed track palaeosurface have an angular, box-shaped cross-section (right image same as left, but shows interpretation of mud crack structures). Note that these are larger crack fillings than those within the tracks (e.g., Figs. 5A and 7C). (F) Vertical invertebrate burrows on bedding planes present as circular pits that are ∼0.6 cm in diameter. White arrows mark the traces. (G) Non-branching, unlined, unornamented, slightly curving to straight, horizontal, cylindrical burrows of invertebrates are filled with massive, very fine grained sandstone. White arrows mark the traces that have a constant diameter along their length.

**Figure 4:**
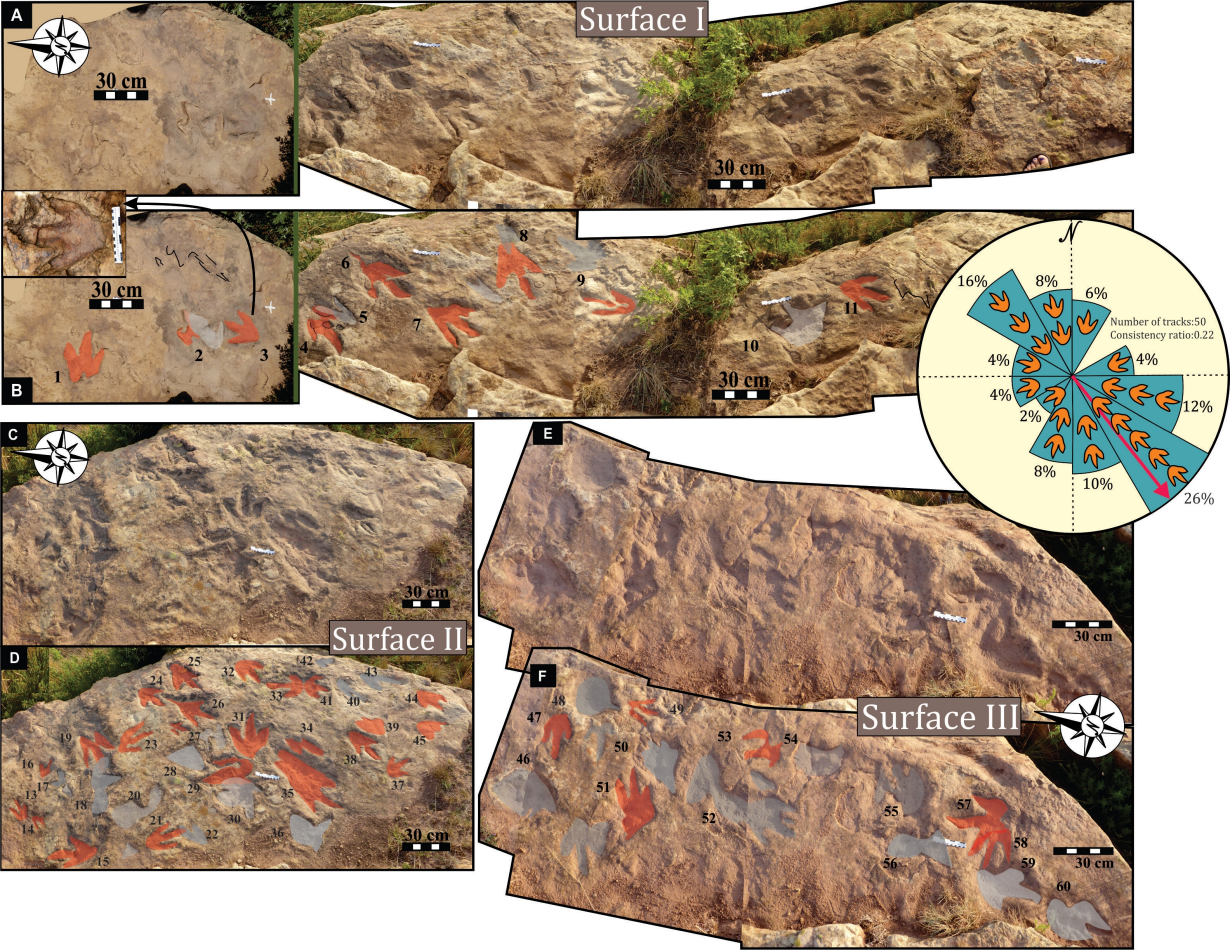
Field views of the Mafube dinosaur track site. Photographs (A, C, E) and their respective interpretative drawings (B, D, F) of Surfaces I, II, and III on the lowermost track palaeosurface with Surface I being the northern-most and Surface III being the southern-most surfaces, respectively. Tracks with reasonably well-defined outlines are shaded in red, and depressions that might represent additional tracks are shaded in grey, and black outlines show incomplete tracks. Inset shows the rose diagram generated from the orientation measurements of 50 tracks, and indicate a mean walking direction to ∼SE (143°), with and angular deviation of ±72°, a vector magnitude of ∼11 and consistency ratio of 0.22.

**Figure 5:**
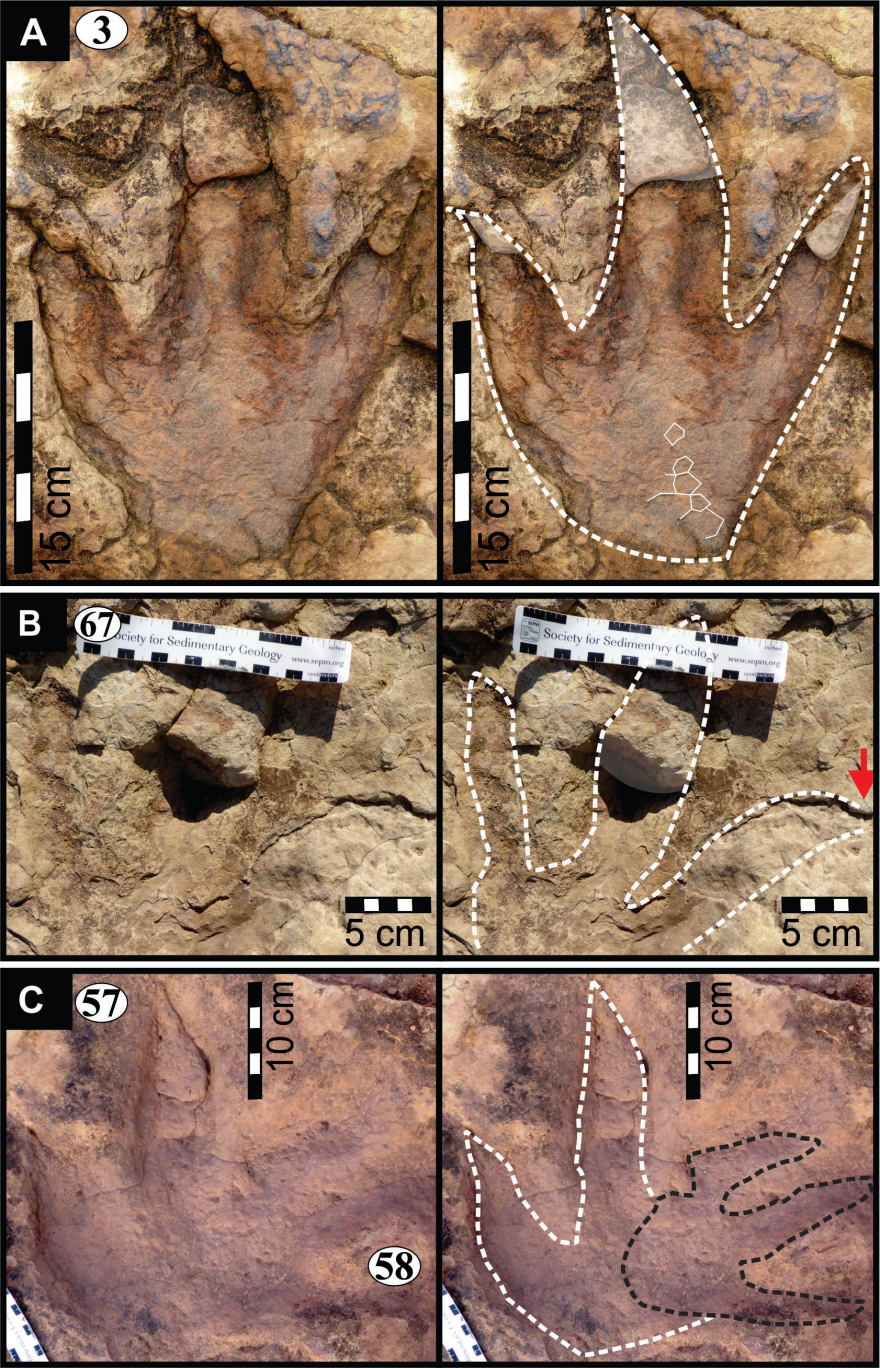
Photographs and interpretative outline tracings of individual tracks (morphotype A) at the Mafube dinosaur track site. (**A**) Track*#3* is one of the best preserved tracks with steep track walls and a clearly defined heel. The desiccation cracks within the foot impression indicate that it is a true track. Note the natural cast at the tips of digits II and IV, the claw mark on digit II and slightly curved, V-shaped digits. (**B**) Track #67 shows sandstone natural casts filling digit III and a claw mark on the rightly-curved digit IV (red arrow). (**C**) Overlapping tracks #58 and 57 point in different directions with angle of difference between digits III being 78°. Note the pitted textures (4 mm in diameter) in track #57 and three rounded, straight digits associated with track #58.

**Figure 6:**
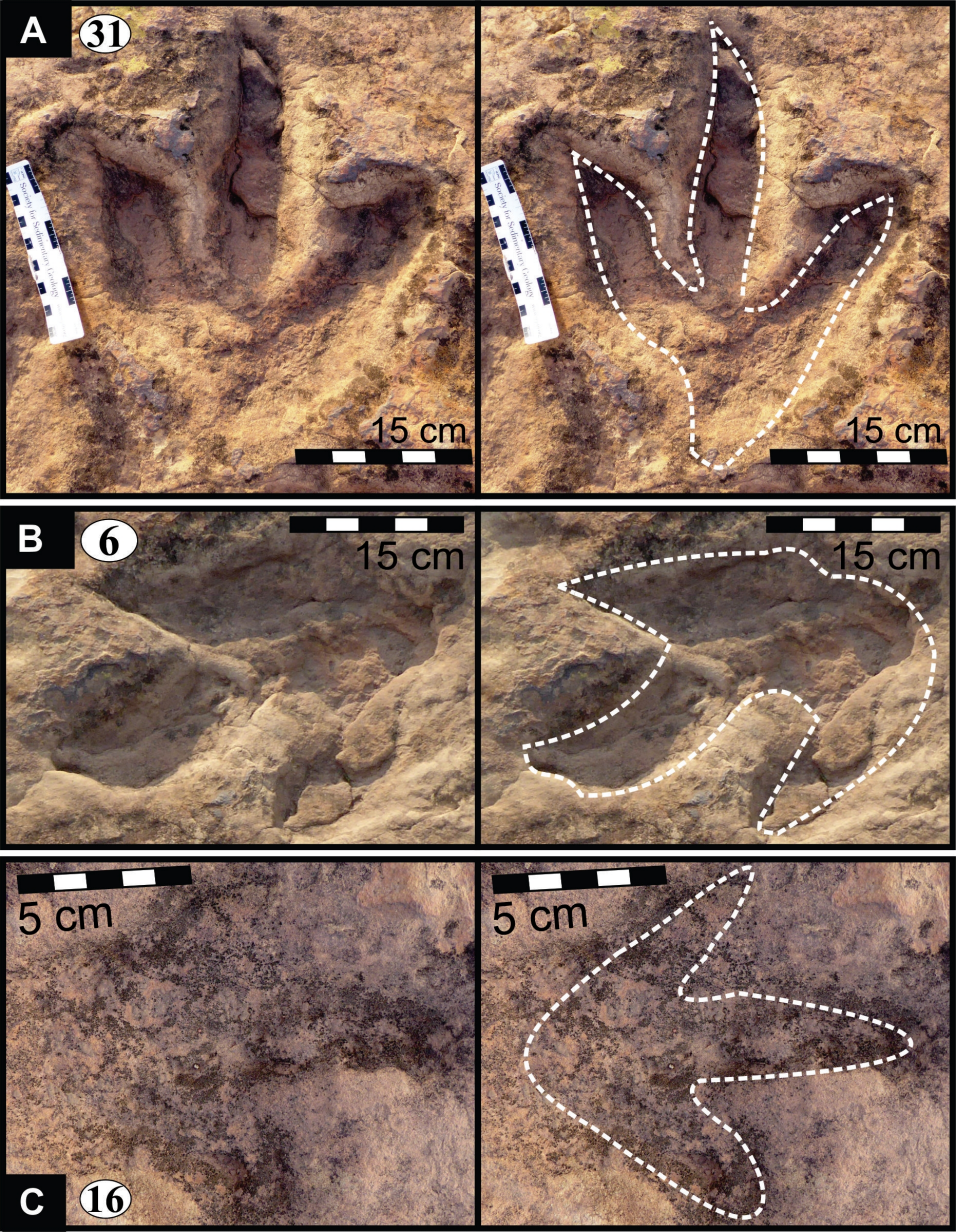
Photographs and interpretative outline tracings of individual tracks at the Mafube dinosaur track site. (A) Right pes of track #31 represents one of the largest tracks at Mafube (Fig. 5C; Table 1) with its digit II alone being 21 cm long. The digit impressions have a distinct curvature towards their V-shaped tips; digit II and IV curve divergently. Digit III retains some of its natural cast. The digit impressions are deeper than the poorly defined heel. (B) Track #6 has steep track walls and V-shaped digits with their natural cast still largely present at their tips. (C) Track #16 represent morphotype B as it is half the size of the larger tracks of morphotype A. This very shallow track shows straight digits with U-shaped tips.

**Figure 7:**
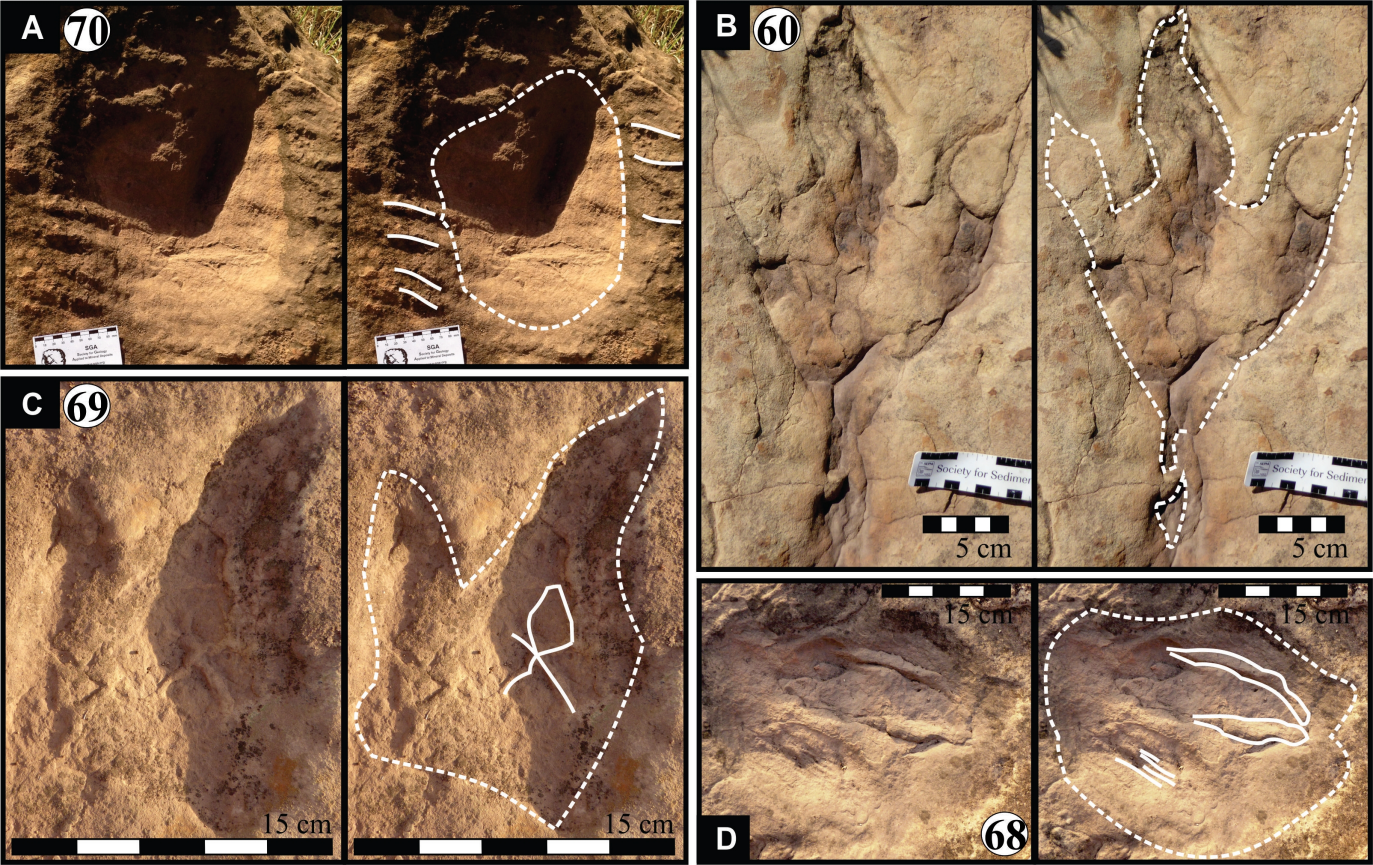
Isolated modified true tracks. (A) Track #70 is covered by well-preserved asymmetrical ripple marks (height: <1 cm; wavelength: 1.6 cm) indicate that a unidirectional water current gently washed over the palaeosurface after the track was made. (B) Track #60 is associated with a poorly preserved impression of the metatarsal. Note the claw marks on all three digits. (C) Track #69 is a true track in spite its amorphous morphology and preserves casts of desiccation cracks indicative of subaerial exposure subsequent to track formation in a moist substrate. (D) Track #68 illustrates how the wet pliable, plastic sediment was squeezed between the digits and collapsed around the track resulting in an indistinct, amorphous morphology. Ridges in the impression of digit IV probably resulted from the accidental skidding of the animal’s foot over a water-saturated sandy substrate (cf. Milàn, 2006).

**Figure 8:**
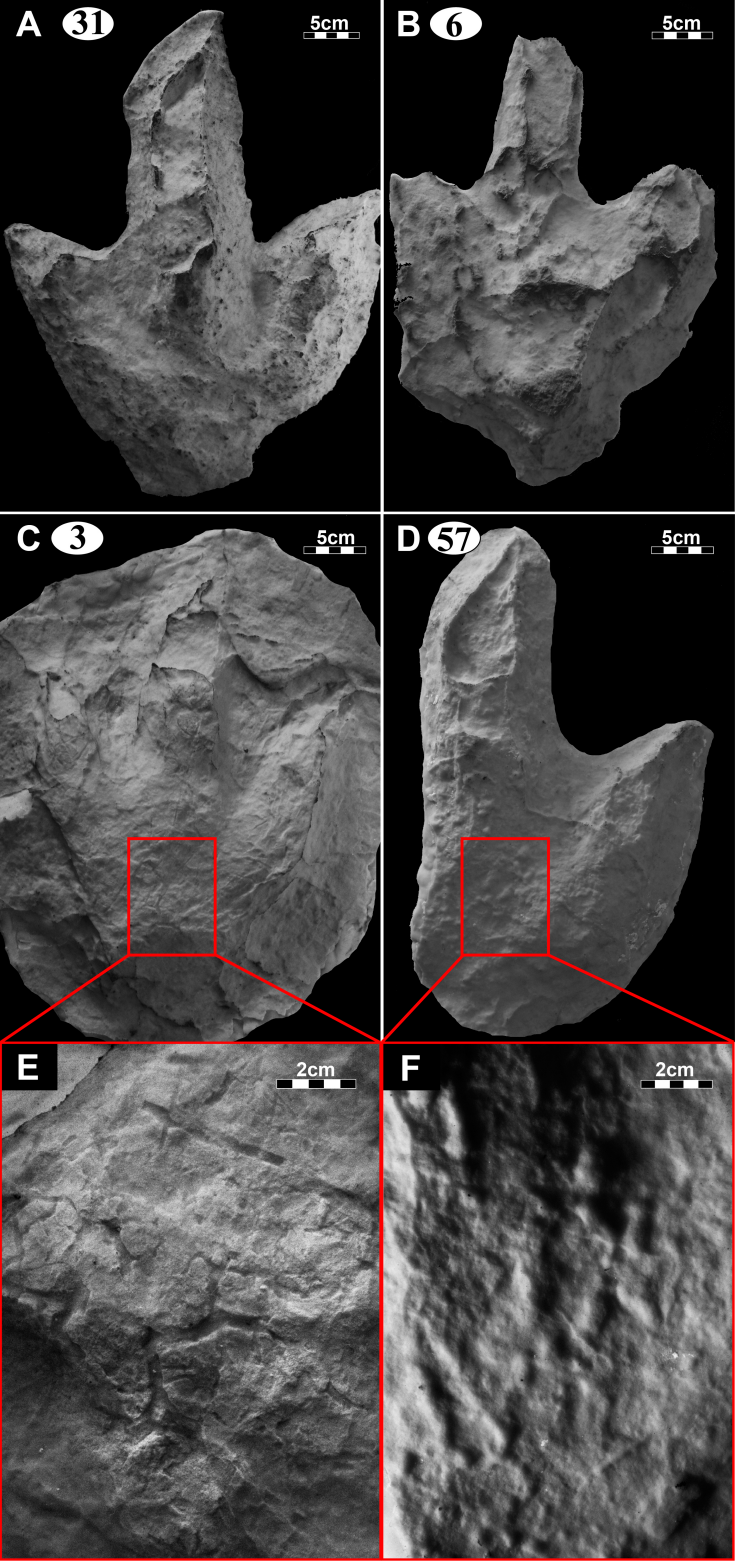
Casts of the best preserved tracks at the Mafube dinosaur track site. (A) Cast of track #31 on Surface I. (B) Cast of track #6 on Surface I. (C) Cast of track #3 on Surface II, with corresponding close-up photograph (E) of the desiccation cracks. (D) Cast of track #57 on Surface III, with corresponding close-up photograph (F) of the pitted texture.

The Mafube tracks are preserved in a 300 m long, 2 m thick, very fine grained, yellow to cream-coloured, tabular sandstone unit within the uEF (Fig. 1). Internally, this host sandstone unit consists of up to ten distinct tabular sandstone beds each 20–25 cm thick. The last three sandstone beds are separated by exposed palaeosurfaces that contain vertebrate tracks, invertebrate traces and casts of desiccation cracks (Figs. 3–8). The lowermost palaeosurface, with an exposed surface area of ∼1 m by ∼20 m, approximately 40 cm below the top of the sandstone unit contains the most numerous and best-preserved tracks at Mafube and is therefore the focus of this study.

The primary sedimentary structures in the sandstone unit are horizontal laminations, low-angle crossbedding (Figs. 3A and 3D), ripple cross-lamination, ripple marks (Fig. 7A) and massive beds. Horizontal lamination is the dominant primary sedimentary structure and low-angle cross-bedding is more commonly observed than ripple cross-lamination (Figs. 3A, 3D and 7A). Secondary sedimentary structures penetrating into the sandstone unit are casts of desiccation cracks and bioturbation (Figs. 3E–3G). The primary sedimentary structures are locally disrupted by soft sediment deformation features, the majority of which are expressed as wavy structures of variable length (Figs. 3B and 3C). Some of these are considered undertracks, because of the distinct concave-up bending of the strata directly underneath tracks on the palaeosurface (Fig. 3C).

**Table 1:** Anatomical measurements of key tracks discussed in text relating to the size differential between morphotypes A and B at the Mafube dinosaur track site. See Fig. 2B for how the interdigit (i.e., divarication) angle, length (*t*), width (*w*), length of rear of phalangeal part of foot (*r*) have been measured in this study (cf. Olsen, Smith & McDonald, 1998). All distance measurements are in centimetres; angles are in degrees. N/A: Measurements could not be determined due to e.g., absence of digit impressions; absence of heel impression; presence of the natural cast obscuring the track See Table S1 for additional track measurements.

**Footprint #**	**3**	**7**	**16**	**27**	**31**	**57**	**58**	**60**	**67**	**68**	**69**	**70**
Length	33.2	34	16.6	16.9	33.4	33.2	N/A	25.4	24.73	27	N/A	28
Width	21.8	24.9	10.8	11.8	26.3	N/A	N/A	21.4	N/A	N/A	N/A	21
Ratio of length/width	1.52	1.37	1.53	1.43	1.27	N/A	N/A	1.19	N/A	N/A	N/A	1.33
*r*	21.2	20.2	9.2	9.4	22.5	20.1	N/A	17.8	N/A	N/A	N/A	N/A
Interdigit angle II^∧^IV	55.1	66.7	56.1	67.8	65.91	N/A	N/A	57.7	N/A	N/A	N/A	N/A
Hypex shape II^∧^III	V	U	V	V	V	U	Wide U	N/A	U	N/A	N/A	N/A
Hypex angle II^∧^III	43	N/A	72.59	N/A	46	N/A	N/A	N/A	N/A	N/A	N/A	N/A
Interdigit angle II^∧^III	24.8	35.91	29.94	35.06	30.13	35.28	N/A	N/A	N/A	N/A	N/A	N/A
Hypex shape III^∧^IV	U	V	V	V	V	N/A	Wide U	N/A	V	N/A	V	N/A
Hypex angle III^∧^IV	32	37.48	71.57	N/A	65	N/A	N/A	N/A	N/A	N/A	N/A	N/A
Interdigit angle III^∧^IV	30.3	30.8	26.16	32.75	35.78	N/A	N/A	N/A	N/A	N/A	35	N/A
Morphotype and Remarks	A	A	B	B	A	A	A	A; metatarsal impression	A	?A; Sediment collapse	?A; Desiccation cracks	?A; Ripple marks
Figure # in text	4A, 5A; 8C	4A	4B; 6C	4B	4B, 6A, 8A	4C, 5C; 8D	4C, 5C	4C, 7B	5B	7D	7C	7A
	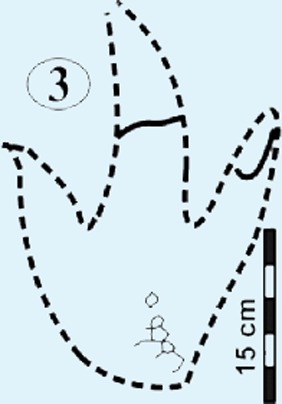	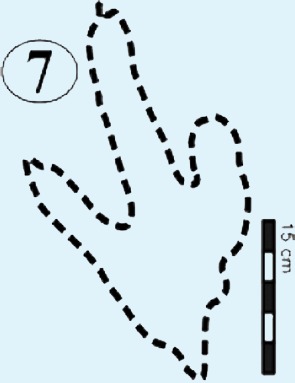	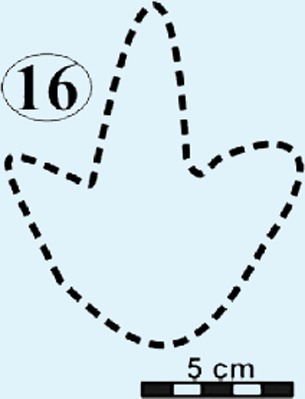	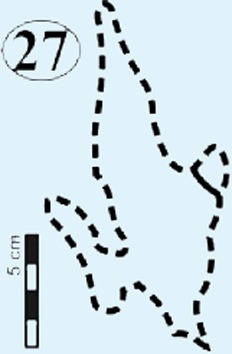	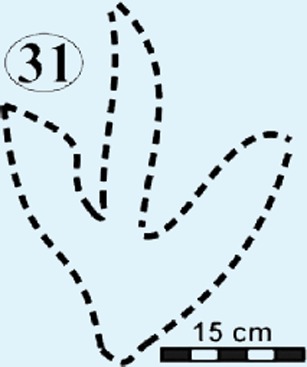	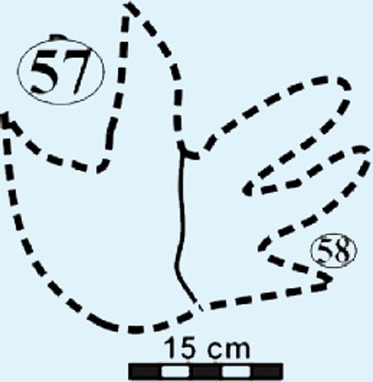		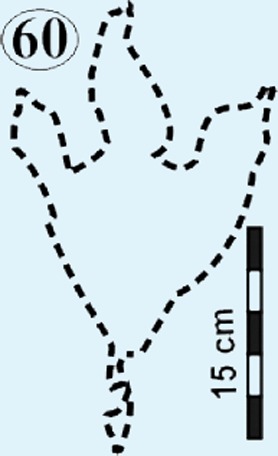	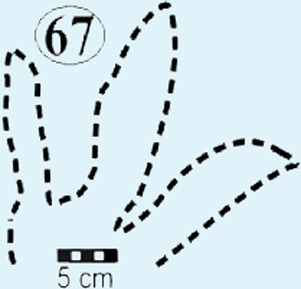	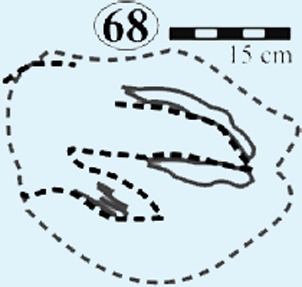	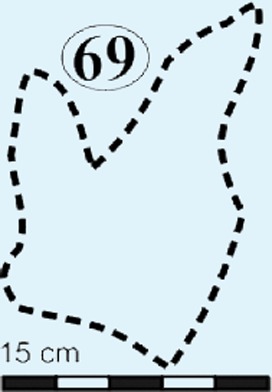	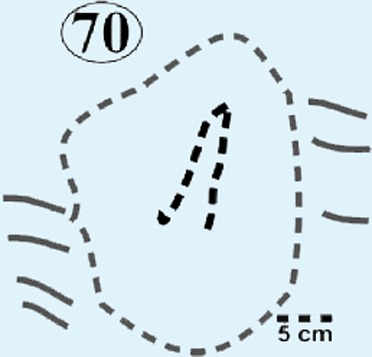

Hexagonal casts of desiccation cracks that penetrate into the sandstone are preserved on the exposed track palaeosurface (Figs. 3E and 3F) and on the surface of some tracks (Figs. 5A and 7C). The casts of desiccation cracks are larger on the track surface (maximum diameter of 11 cm) than the smaller casts of desiccation cracks within the tracks (maximum diameter of 2.5 cm). Bioturbation on the track palaeosurface is due to invertebrate burrowing that comprises of cylindrical tubes that are non-branching, unlined, unornamented, and have uniform diameter along their length. The burrows are both horizontal and vertical (Figs. 3F and 3G) and lack internal structures (i.e., are filled with massive, very fine grained sandstone). The horizontal invertebrate traces are slightly curving to straight, ∼0.5 cm in diameter, and range in length from ∼3 to ∼22 cm. Surface texturing within some of the bedding plane depressions and tracks (Fig. 5C) comprise pits with variable diameters ranging from a few mm to 1 cm with a mean pit diameter on a mm-scale (∼4 mm). The subdued relief of the pits prevents more accurate measurements.

### Description of the track site

Over 80 tracks have been identified at the Mafube dinosaur track site (Fig. 4) over a lateral distance of ∼80 m. The degree of track preservation varies and some of the better preserved tracks show claw marks as well as desiccation cracks, ripple marks, etc. (Figs. 5–8). Based on key biological and sedimentological features the lowermost palaeosurface is sub-divided into three surface tracts, named: Surface I, II and III (Fig. 4). There are additional tracks preserved on the same lowermost palaeosurface, but they are sparse and far apart (Fig. 7). Directional information obtained from 50 tracks of the palaeosurface is presented in a rose diagram (inset in Fig. 4). The orientation of the tracks is multi-directional and no individual trackways are recognised on the palaeosurface

#### Surface I

This is the northern surface of the Mafube site (Figs. 4A and 4B). Twelve tracks are preserved on the 4.5 m long Surface I, three of which do not provide meaningful morphological or directional information. Except for the poorly preserved tracks (e.g., track #2, 5, 10) the tracks show three digits and have similar morphologies. Several tracks on Surface I have *V*-shaped hypices with lengths constrained between ∼25–35 cm and widths of ∼20–30 cm. The morphology of the digit tips is somewhat obscured by the sandstone natural cast but appears to be predominantly *V*-shaped. Tracks #3, 6, 7 and 11 (Fig. 4A) are the best preserved on Surface I. Track #3 (right footprint) is one of the best preserved tracks at the Mafube site and shows claw marks and casts of desiccation cracks (Fig. 5A). Track #4 displays claw marks and track #11 contains only casts of desiccation cracks (Figs. 4A and 4B).

#### Surface II

This is the middle track palaeosurface of the Mafube site (Figs. 4C and 4D). A total of 33 tracks are preserved on the 3.8 m long Surface II. Seven of these tracks do not provide meaningful morphological or directional information. There is a large size variation in the 33 tracks which range between ∼13–36 cm in length and ∼10–28.5 cm in width. Tracks #13, 14, 16 and 27 appear to have been made either by juveniles or a different, smaller species than the larger trackmaker (e.g., track #31). These smaller tracks have an average length and width of 13 cm and 10 cm, respectively, and define morphotype B at the Mafube site. All other tracks belong to morphotype A. The best preserved large track #31 (right footprint; Figs. 4D and 6A) has an exposed length of 36 cm and a width of 28.5 cm. Track #31 is also noteworthy for its claw marks on digits II and III and distinct curvature to digit III (Figs. 4D and 6A). A potential claw mark is also seen in Track #38 but the presence of the natural cast in the foot impression makes its assessment difficult (Fig. 4D). Although tracks #33 and 41 are marked separately, there is some degree of overlap in the heel region (Fig. 4D).

#### Surface III

This is the southern surface of the Mafube site (Figs. 4E and 4F). Fourteen tracks are preserved on the 3.2 m long surface, with only five tracks showing discernible morphological and directional information. No trackways can be identified on Surface III. Overall morphologies are poorly defined and do not allow for accurate measurements. However, Surface III is important because of the pitted textures in the tracks themselves but which are absent on the palaeosurface between adjacent tracks. This texture is most obvious in tracks #52, 57 and 58 (Figs. 4E and 4F).

#### Description of selected tracks

The summary below accounts for the key characteristics of the best preserved large (e.g., #3, 31, 57, 58, 67 of morphotype A) and small (#16 of morphotype B) tridactyl tracks as well as tracks of particular interest (#60, 68, 69, 70; see Figs. 4–8 for illustrations and Table 1 and Table S1 for measurements).

*Track #3*, a right footprint is one of the best preserved tracks and shows desiccation cracks indicating that it is a true track (Fig. 5A; cast in Fig. 8C; Table 1). Moreover, the track has a natural cast at the tips of digits II and IV, a 2.5 cm long claw mark on digit II and slightly curved, *V*-shaped digits, especially in case of digit II. The track walls are steep and the heel is clearly defined.

*Track #67* retains most of its natural cast, therefore the depth of the impression is unknown (Fig. 5B; Table 1). A claw mark on the rightly-curved digit IV is present.

*Tracks #57 and 58* are superimposed on one another (Fig. 5C) and point in different directions (angle of difference between digits III is ∼78°). Track #57 lacks digit IV (Fig. 8D; Table 1) and so its total width is unknown. Digits II and III curve slightly towards the tips. Pitted textures (∼4 mm in diameter) are observed in track #57 (Figs. 8D and 8F). The less clearly defined, shallow impression (not suited for length and width measurements) associated with track #58 preserves three rounded, straight digits.

*Track #31* is one of the largest tracks at the Mafube site, a right pes (Fig. 6A; Table 1) with its digit II alone being 21 cm long. The digit impressions are deeper than the poorly defined heel and are relatively straight with distinct curvature towards their *V*-shaped tips. Curvature of digit II and IV is divergent. Steep track walls surround digit III which retains some of its natural cast.

*Track #6* is a left pes which forms a depression of variable depths because its natural cast is still largely present (Fig. 6B; Table 1). All three digits are V-shaped. The cast (Fig. 8B) was made with leftover casting mixture which did not fully capture all the properties of the track, thus limiting accurate measurements.

*Track #16* is half the size of the larger tracks being only 16.6 cm long and 10.8 cm wide (Fig. 6C; Table 1). This small and very shallow impression has straight digits with U-shaped tips.

*Track #70* is isolated, morphologically indistinct and preserves ripple marks consisting of very fine grained sandstone (Fig. 7A; Table 1). The small ripple marks are asymmetrical and <1 cm high with a wavelength of 1.6 cm.

*Track #60* preserves claw marks and a poorly preserved impression of the metatarsal (Fig. 7B; Table 1). Claw marks are preserved on all three digits and terminate in very acute angles. The natural cast obscures numerous morphological characteristics (e.g., shape of digits and hypex; inter-digit angles).

*Track #69,* a modified true track, preserves casts of desiccation cracks with desiccation polygons that range in diameter from 1.9–2.7 cm (Fig. 7C; Table 1). Although the indistinct morphology of the track prevents accurate length and width measurements, the following morphological characteristics can be observed: (1) impressions of digits II (?) and III; (2) the increasing depth of the impression towards the heel; (3) slight upward and downward curvature of digit III and IV, respectively; (4) *V*-shaped hypex; and (5) interdigit angles of 35°.

*Track #68* lacks distinct morphology, however digits III and ‘ridges’ within digit II or IV (digit cannot be adequately identified) may be inferred (Fig. 7D; Table 1). Sediment-collapse features include the semi-parallel ridges surrounding *U*-shaped digit III as well as ridges in the impression of digit IV.

**Figure 9:**
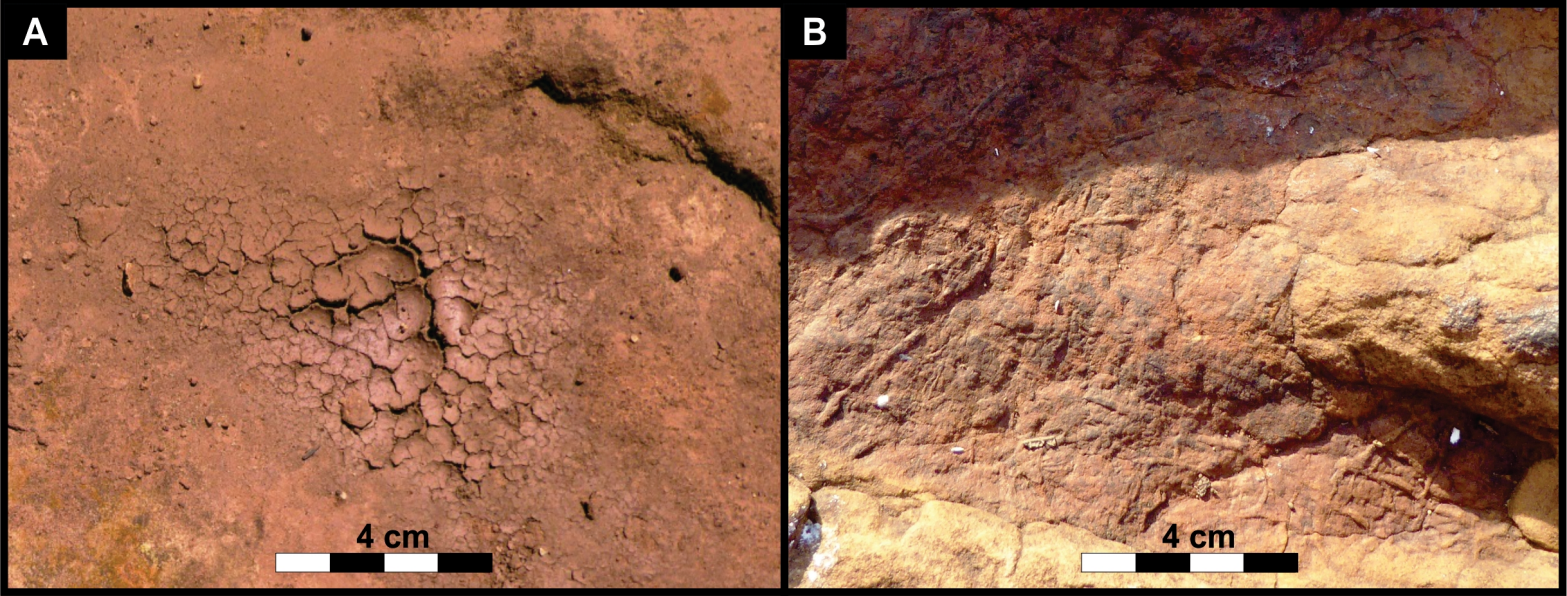
(A) Modern desiccation cracks within a thin, mud layer in the depression of an Lower Jurassic track at the study site. (B) Casts of ancient desiccation cracks that had been preserved inside the Lower Jurassic track. The cracks are comparable in size and shape, having a characteristic hexagonal cracking pattern. The modern example formed after a summer thunderstorm following the evaporation of the muddy storm water from the track depression. The subaerially exposed mud layer cracked up due to dewatering within a few hours. These cracks may then be filled with sandy sediment in subsequent sedimentation events to form similar casts as in tracks #3 and 69.

## Discussion

### Genesis and preservation of the track surface

Interpretations of the high energy, flash-flooding and episodic drying out of the stream/river channels in the uEF (Bordy, Hancox & Rubidge, 2004) are well-illustrated at the Mafube dinosaur track site. It preserves low energy sedimentary structures (bioturbated, desiccated and cracked palaeosurfaces) that overlie and are separated from upper flow regime, very fine grained horizontally laminated sandstones. Ephemeral streams/rivers typical of semi-arid environments are also characterised by fairly sudden reductions in the volume of running water (due to evaporation and seepage), transforming them into very shallow ponds or pools with still-standing or slow moving water. It is within these stagnant water bodies that sediments may be rippled and colonized by microbial organisms and other invertebrates before desiccating. Sedimentary structures on the track surface show that the tracks were made in a dense, but soft, water-saturated sediment and, in some places, below the surface of a shallow water layer. This is because modified tracks show small, asymmetrical ripple marks, soft sediment deformation features (e.g., expulsion rims sediment-collapse structures), invertebrate burrows, and casts of desiccation cracks (Figs. 3–8).

Asymmetrical ripple marks within and around track #70 suggest that some impressions were likely made in an underwater substrate over which gentle, unidirectional currents moved (Fig. 7A). Moreover, the cast of desiccation cracks on the track palaeosurface (e.g., Figs. 5A and 7C) suggest that the sediment surface was covered in a thin, wet mudfilm that dried out and was ultimately covered by sandy sediments that also infilled the cracks and formed a natural casts (Fig. 9). The smaller desiccation cracks within tracks indicate that track depressions (e.g., tracks #3, 69 in Figs. 5A and 7C) were able to collect muddy floodwater. Upon evaporation, the muddy floodwater left behind a thin, mud-cracked sediment veneer similar to that observed in a modern track after a summer thunderstorm (Fig. 9).

Overall, the sedimentary structures preserved in and around the tracks show that the exposed surface is largely the original one on which the theropods moved, essentially contemporaneously, in the Early Jurassic. Therefore, the best-preserved tracks are faithful replicas of the anatomical features of the feet of these Early Jurassic dinosaurs. Taxonomically less valuable undertracks (e.g., Fig. 3C) have also been identified in the form of deformation features in the sediment layers below the original track surface. Such features are a combined result of the size and locomotion style of the trackmaker (e.g., large running adult) as well as the rheological properties of the sediment (i.e., sediment consistency) on which the animals moved (e.g., very fine grained, plastic, water-saturated sand layer) (Gatesy, 2003; Milàn, 2006; Milàn & Bromley, 2006).

From north to south, the tracks on the surfaces become less morphologically distinct resulting in a track morphology that is highly variable over the length of the study site. For instance, several tracks of similar size show variable depths of imprinting (e.g., tracks #3, 26, 60, 69 in Figs. 4, 5A and 7C). Furthermore, the amorphous quality of tracks on Surface III (Figs. 4E and 4F) is likely due to the different consistency of the substrate in that area of the palaeosurface (e.g., more water saturated conditions). The faint, amorphous nature of the isolated tracks within the southern portion of the site further south of Surface III may also be due to sediment-collapse features that obscured the original track as the substrate was too wet to allow the production and preservation of well-defined tracks. Ripple marks, expulsion rims and other soft sediment deformation features resembling squish marks (Fig. 7) are only observed together with the isolated tracks found in the southern section of the site. In contrast, tracks express more detail (e.g., claw marks, desiccation cracks; track #3 in Figs. 4A, 4B and 5A) in the north. This trend in track preservation with tracks to the south having a generally lower degree of anatomical preservation and higher abundance of associated soft sediment deformation features (such as expulsion rims, e.g., tracks #31 and 68 in Figs. 6A and 7D, respectively) is evidence for the changing consistency of the substrate. This is specifically indicative of an increasingly moist substrate along the palaeosurface to the south, possibly due to the proximity of a water pool. The preservation of metatarsal impressions (track #60 in Fig. 7B) are likely related to the animals’ movement in the more water saturated substrate (cf. Gatesy, 2003). The presence of metatarsal impressions and slipping tracks (e.g., tracks #60, 68 on Figs. 7B and 7D) on Surface II reinforces the interpretation of a water saturated substrate.

Despite the lack of trackways within the multi-directional tracks at Mafube, when quantified (inset in Fig. 4), the track orientations reveal a dominant movement direction to the south-east and a less dominant one to the north-west. Locomotion towards the south-east is compatible with the position of a potential water source (e.g., ephemeral water body) to the south-east of the palaeosurface as indicated by the sedimentological and track taphonomic results. Therefore, it may be assumed that the tracks indicate animal movement towards this water source for drinking and/or feeding purposes, and that not all animals returned along the same route (to the north-west). Furthermore, the close spacing and multi-directionality of the tracks (Fig. 4) suggest a moderate degree of trampling especially in the area of the track palaeosurface shown in Figs. 4C and 4D.

**Figure 10:**
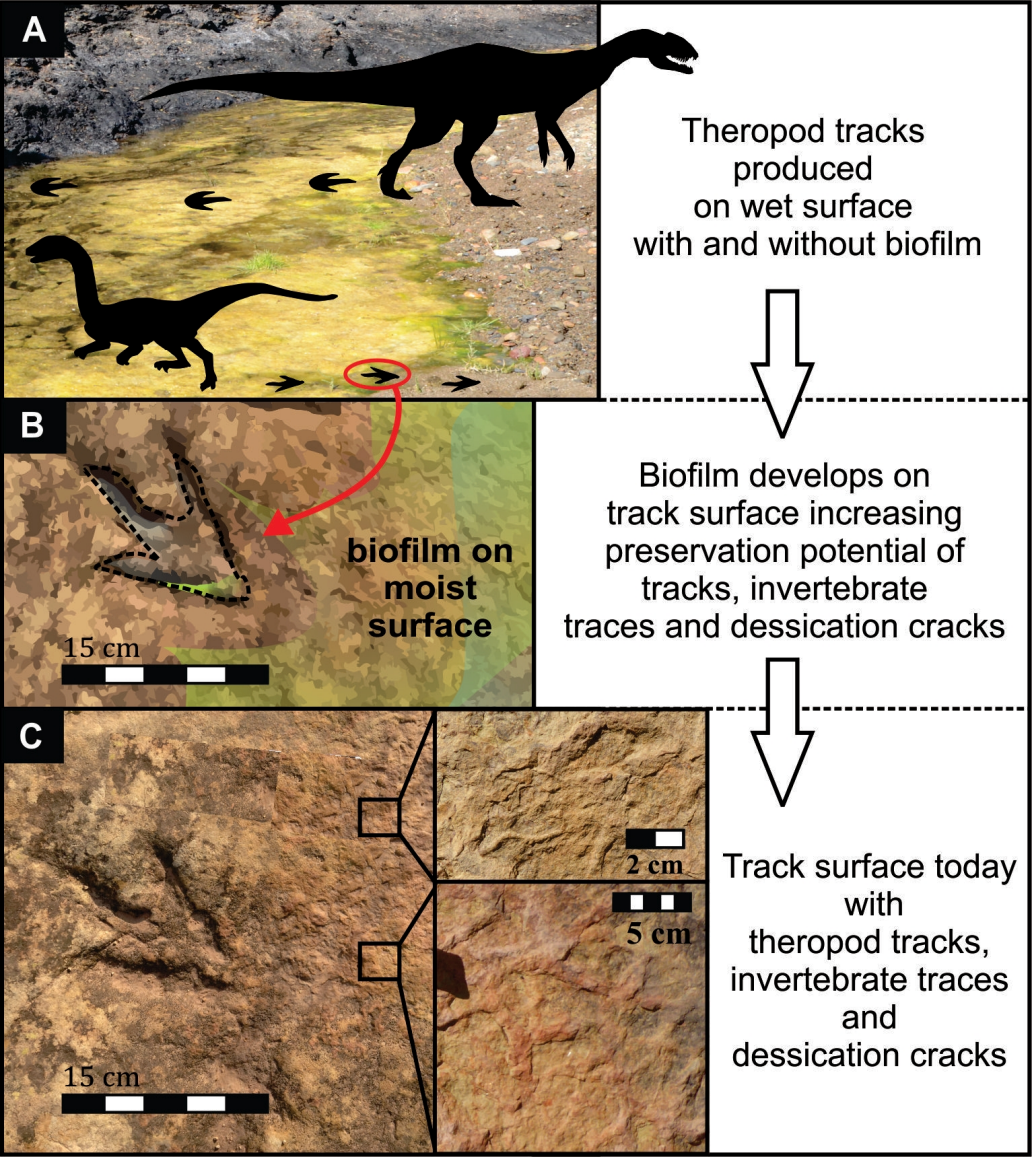
Model of the formation and preservation of tracks at the Mafube dinosaur track site. Not to scale. *Dracovenator* outline adapted from https://commons.wikimedia.org/wiki/File:Dracovenator3.png.

Events following the formation of a track are crucial for its preservation, because exposed tracks are highly degradable via erosional processes acting on the sediment surface (e.g., wind or water currents). Therefore, at Mafube, the solidification of the imprinted layer had to be relatively rapid, because the deposition of the overlying sediment layer occurred without producing erosional features. Instead, during the deposition of the overlying sediment layer, sandy sediment infilled (and formed natural casts in) the delicate desiccation cracks and depressions between ripple marks as well as theropod foot impressions. Given that the sedimentary structures in the overlying very fine grained, horizontally laminated sandstones indicate upper flow regime conditions, the pervasive solidification of the palaeosurface was likely enhanced not only by subaerial exposure (i.e., moisture evaporation), but potentially also by microbial mats that biostablised the granular, sand-rich sediment layer, and ultimately produced a more cohesive and thus less erodible substrate (cf. Carvalho, Borghi & Leonardi, 2013; Fig. 10). The pitted texture in Surface III (Figs. 4E, 4F, 5C, 8D and 8F) somewhat resembles both experimentally produced biofilm covered surfaces (e.g., Mariotti et al., 2014) and “wrinkled textures” described by Parizot et al. (2005; see their Fig. 15), Schieber et al. (2007) and Mariotti et al. (2014; see their Fig. 3A) which have been interpreted as the remnants of microbial mats reinforcing sandy, granular substrates. The partially subaqueous nature of the Mafube palaeosurface (explained in detail below) may have supported microbial growth, especially in the tracks that may have acted as pools for stagnant water (Fig. 10). These puddles would have allowed for localized algal blooms despite rapid drying or absorption on the bedding surface as a whole. Nonetheless, the quality of the pitted texture is poor and variable within and between tracks and therefore it is hard to show conclusively that microbial mats were widespread or even present at Mafube. All in all, there is no credible evidence to wholly support this texture as being part of a microbially induced sedimentary structure, and at best is should be characterised as a sedimentary surface texture sensu Davies et al. (2016).

### Identity of the possible trackmaker

Tracks found to date at the Mafube are tridactyl, barring several which are poorly preserved (e.g., Track #69) giving the impression of being didactyl. The anatomical detail of the tracks decreases towards the southern end of the site. The lack of manus impressions is indicative of obligate bipeds and eliminates quadrupedal trackmakers. We link the Mafube tracks to theropods based on the following criteria that distinguishes tracks of tridactyl theropods from ornithischians: (1) long, slender digits, that are asymmetrical, taper; and (2) often end in a sharp claw impression or point (e.g., track #3 in Figs. 4A, 4B and 5A); and (3) the tracks being longer than broad (Olsen, Smith & McDonald, 1998; Raath & Yates, 2005).

If the interpretation of the Mafube tracks being made by large and small theropod dinosaurs is correct they can then be compared to body fossils of theropods within the uEF and Clarens Formation in South Africa and Lesotho. To date, there are only two known theropod dinosaurs capable of producing such tridactyl tracks in the Lower Jurassic geological record of the southern Africa: *Dracovenator regenti* (Yates, 2005) and the coelophysid theropod, *Coelophysis rhodesiensis* (formerly *Syntarsus rhodesiensis* Raath, 1977; Bristowe & Raath, 2004). The latter is known from the Lower Jurassic Karoo-aged deposits in Zimbabwe (Raath, 1977), whereas fragmentary remains of *Coelophysis* sp. have been identified in the uEF of South Africa (Kitching & Raath, 1984; Munyikwa & Raath, 1999). Globally, the type species of *Coelophysis* (*Coelophysis bauri*) and an anomalously high abundance of specimens are known from the Upper Triassic Chinle Formation (Schwartz & Gillette, 1994) and remains have also been reported in the Kayenta Formation (Glen Canyon Group; Rowe, 1989) and Moenave Formation (Lucas & Heckert, 2001) in the Lower Jurassic of the USA.

### Potential ichnotaxa

The Mafube track surfaces preserve tridactyl tracks which can be distinguished on the basis of their size. The commonly occurring morphotype A has a length averaging 32 cm in contrast to rare morphotype B which has a length between 10–13 cm (e.g., tracks #16, 47 in Figs. 4C, 4D and 6C). Both Mafube morphotypes can be attributed to theropod trackmakers. Morphotype A represents medium to large animals and morphotype B represents smaller animals or juvenile individuals of the larger trackmaker. Given the track morphology, only two theropod dinosaur ichnotaxa are possible candidates for the tridactyl tracks at Mafube. Morphotype A may be related to the ichnotaxon *Eubrontes* based on morphological characteristics while morphotype B may be considered more representative of the ichnotaxon *Grallator*.

#### Morphotype A

Eubrontes-like tracks are digitigrade and tridactyl with narrow interdigit divarication angles and *V*-shaped hypices between digits. Several tracks show a poorly preserved metatarsal impression (track #60 in Fig. 7B). Track length to width (t/w) ratios average 1:3. The rear margin of the tracks often displays an indentation, which would be associated with a heel-like bulge from the proximal end of digit IV which aided with left or right pes identification (e.g., track #69 in Fig. 6B (Da Silva et al., 2012). Hypices are normally *V*-shaped and only occasionally *U*-shaped between digits II and III (e.g., tracks #67, 6 in Figs. 5B and 6B, respectively). The divarication angles average 30°between digits II and III and 35°between digits III and IV.

Importantly, there is some variation in the size of morphotype A which ranges from 24 to 34 cm in length and from 17 to 31 cm in width (Table 1; Table S1). Sediment consistency (increasing/decreasing visco-elasticity) may result in a wide range of track dimensions as the foot is pulled through the medium, but large variations in size (here nearly 50%) are not expected to be due to this abiotic control only. The combined effect of substrate characteristics variation in foot shape and size and associated limb dynamics most likely produced the wide range of track morphologies at this site (see Pèrez-Lorente, 2015, p. 176, 267).

Morphotype A is comparable to medium-size *Eubrontes* tracks that are >25 cm in length, bipedal and tridactyl with short digit III and a broad track width as well as outer digits that diverge at an angle which varies between 25–40° (e.g., Rainforth, 1997; Olsen, Smith & McDonald, 1998). These features all compare favourably with the measurements taken from several well-preserved examples of Mafube morphotype A (Table 1). *Gigandipus* is another ichnotaxon to which these larger Mafube tracks bear some resemblance based on their size and occasional preservation of a metatarsal impression although no tail drag traces were observed at Mafube (Rainforth, 2004; Rainforth, 2005). The ichnogenus *Gigandipus* has been considered, along with *Anchisauripus*, to be synonymous with* Eubrontes* (Milner, Lockley & Johnson, 2006 –see further discussion below).

#### Morphotype B

Grallator-like tracks are also digitigrade and tridactyl with narrow interdigit divarication angles and *V*-shaped hypices. The track length to width (t/w) ratio averages 1.0 for the six better preserved specimens.

The size of morphotype B (length ∼15 cm) is comparable to those tracks that were described as *Grallator* by Olsen, Smith & McDonald (1998) which are ≤15 cm long, bipedal, tridactyl and with digit III projecting considerably anteriorly. The ichnogenus *Grallator* occurs globally (e.g., in the Fundy Basin (Canada), Hartford, Deerfield and Newark basins of USA by Olsen & Galton, 1984; Olsen, Smith & McDonald, 1998; Lucas et al., 2006; in Australia by Thulborn, 1998; in France by Gand et al., 2005; in China by Lockley & Meyer, 1999 and can be fairly reliably linked to small theropod dinosaurs with a foot length of <25 cm (Thulborn, 1990).

Compared to *Eubrontes*, *Grallator* impressions are narrower with a length to width ratio ≥2 and the outer digits diverge at an angle of 10–30°(Olsen, Smith & McDonald, 1998; Lucas et al., 2006). Generally, *Grallator* is characterised by the preservation of pad and claw impressions (Rainforth, 1997). These key features which define *Grallator* are similar to the smaller track described here (Fig. 5; Table 1; Table S1). However, the poor anatomical details of tracks at Mafube prevent their confident ichnotaxonomic assignment to *Grallator*. Notwithstanding, these rare, *Grallator*-like Mafube tracks could have been produced by *Coelophysis* because the size of the tracks (length <15 cm; Table 1) is comparable to the fossil pes measurements of *Coelophysis* reported by Olsen, Smith & McDonald (1998). Measurements of skeletal remains from both South Africa and North America have phalangeal lengths ranging from 12.8–13.9 cm, with the divarication angle between digits II and IV ranging from 12–17° (Olsen, Smith & McDonald, 1998). Based on these close similarities, morphotype B tracks can be tentatively linked to a theropod trackmaker like* Coelophysis.*

### Comparison to Lower Jurassic theropod tracks of southern Africa

The density, preservation and diversity of Lower Jurassic dinosaur tracks in Lesotho far outnumber other sites in South Africa. Tridactyl tracks ≤35 cm in length have been described by Ellenberger (1970), Ellenberger (1972) and Ellenberger (1974) from the lower and upper Elliot and Clarens formations of Lesotho and been assigned to various ichnogenera such as: Kainotrisauropus isp. Qemetrisauropus isp., Prototrisauropus isp., Deuterotrisauropus isp. However, several authors (e.g., Lucas et al., 2006; D’orazi Porchetti & Nicosia, 2007) consider all these to be synonymous with Eubrontes. Furthermore, Olsen & Galton (1984) synonymized all occurrences of Kainotrisauropus with Grallator.

The larger *Eubrontes*-like Mafube morphotype A shows similarities in size and general morphology with a number of Lower Jurassic tracks in South Africa (Uniondale trackway; Raath & Yates, 2005), Lesotho (Moyeni tracksite; Wilson, Marsicano & Smith, 2009) and Zimbabwe (Ntumbe River, lower Zambezi Valley; Munyikwa, 1996). The five successive tracks forming the Uniondale trackway were considered to be similar to *Grallator* as well as the ichnogenera *Kainotrisauropus* (of Ellenberger, 1970) by Raath & Yates (2005). These Uniondale tracks are morphologically similar to tracks from Ntumbe River (Zimbabwe) which Munyikwa (1996) assigned to *Eubrontes.* Moreover, the recently re-analysed Moyeni tracks in the uEF that were originally referred to as *Neotrisauropus* by Ellenberger (1974) have been incorporated into the ichnogenus *Grallator* by Wilson, Marsicano & Smith (2009). Although the Moyeni tracks are larger (∼28 cm in length) than typical tracks assigned to *Grallator* Wilson, Marsicano & Smith (2009) argued that the tracks are below the maximum lengths of similar tracks from the Newark Supergroup, and considered them to be produced by a theropod similar to *Dracovenator regenti*.

The foregoing is reflective of the on-going ichnotaxonomic debate on Upper Triassic-Lower Jurassic tridactyl, non-avian theropod tracks (e.g., Olsen, 1980; Olsen, Smith & McDonald, 1998; Lockley, 1998; Rainforth, 1997; Rainforth, 2005; Lucas et al., 2006; Lockley, 2010). The difficulty with qualitatively distinguishing these tracks is mainly caused by the simplicity of the track morphology and the relatively conservative foot anatomy of non-avian theropods over most of the Mesozoic. Among others, this ichnotaxonomic discourse was examined by Lockley (1998), who briefly accounted for the various quantitative methods for characterising the morphological differences in tridactyl tracks. Some of these approaches have been gaining momentum in more recent studies (e.g., geometric morphometrics in Castanera et al., 2015).

Currently there are four widely used Lower Jurassic ichnogenera of tridactyl, non-avian theropod tracks (*Grallator*, *Anchisauripus, Eubrontes* and *Kayentapus*) despite the current ichnotaxonomic debate. Several other ichnotaxa are considered either behavioural/preservational variations (e.g., *Gigandipus*) or junior synonyms of the four commonly considered valid ichnogenera above. Size has long been the key separating criteria in the *GrallatorAnchisauripusEubrontes* or GAE allometric plexus (Olsen, Smith & McDonald, 1998; Rainforth, 2005; Petti et al., 2011), and synonymising the three ichnogenera into a single valid ichnogenus (*Grallator* or *Eubrontes*) has been long considered and debated (e.g., Olsen, 1980; Olsen, Smith & McDonald, 1998; Rainforth, 2005; Lucas et al., 2006). The ichnogenera *Kayentapus* and *Anchisauripus* have been incorporated into the *GrallatorEubrontes* spectrum largely because of their intermediate size between the two ichnogenera (Olsen, Smith & McDonald, 1998; Lockley, 1998; Milner et al., 2009). The former, with its wide divarication angles and gracile form has been either synonymised within *Eubrontes* (Lucas et al., 2006) or considered as a valid ichnogenus by several authors based on its morphology (Lockley, 1998; Milner et al., 2009; Lockley, Gierlinski & Lucas, 2011).

The foregoing also highlights the plight of assigning relatively large tridactyl tracks to the *GrallatorEubrontes* spectrum, two ichnogenera that are in essence morphologically similar, except for their size. Because track size is naturally variable its ichnotaxonomic utility in this case is questionable (e.g., Rainforth, 2005; Lucas et al., 2006). For example, considering its average length of ∼32 cm, the Mafube morphotype A could be placed between the Moyeni (length 28 cm) and the Uniondale (length 39.7 cm) tridactyl tracks which are the two current end-members of the *GrallatorEubrontes* spectrum in southern Africa. In spite of this problematic, size-based distinction between the otherwise morphologically similar *Grallator* and *Eubrontes* ichnogenera, we consider the larger Mafube dinosaur tracks (morphotype A) to be more evocative of *Eubrontes* and the smaller morphotype B to be more similar to *Grallator.*

## Conclusions

Sedimentological, taphonomic and ichnological assessments of the >80 tridactyl theropod tracks at Mafube dinosaur track site:

1. Reveal casts of desiccation cracks preserved in impressions of true dinosaur tracks which are valuable for reliable taxonomic assessments;
2. Demonstrate how track shape variability is linked to changes in the substrate consistency along the same palaeosurface (in this case, due to increasing moisture content);
3. Confirm animal movement towards a water source despite the multi-directionality of tracks and lack of trackways on the palaeosurface;
4. Confirm the earlier reconstructions of the Lower Jurassic uEF depositional environment and reinforce the interpretation of a fluvio-lacustrine setting, typified by high energy, ephemeral water courses with flash flood-type discharge under semi-arid climatic conditions;
5. Aid the Lower Jurassic ecological assessments by providing evidence that during the deposition of the uEF theropod dinosaurs of variable size were present and left behind tracks possibly representing the *Eubrontes* and *Grallator* ichnogenera.

##  Supplemental Information

10.7717/peerj.2285/supp-1 Table S1 Additional anatomical measurements of Mafube tracks relating to the size differential between morphotypes A and B at the Mafube dinosaur track site Additional anatomical measurements of Mafube tracks relating to the size differential between morphotypes A and B at the Mafube dinosaur track site. See Fig. 2B for how the interdigit (i.e., divarication) angle, length (t), width (w), length of rear of phalangeal part of foot (r) have been measured in this study (cf. Olsen, Smith & McDonald, 1998). All distance measurements are in centimetres; angles are in degrees. N/A: Measurements could not be determined due to e.g., absence of digit impressions; absence of heel impression; presence of the natural cast obscuring the footprint.

## Additional Information and Declarations

### Data Availability

The following information was supplied regarding data availability: The raw data has been supplied as Table S1.
