# Supplementary material for: Sedimentology and ichnology of the Mafube dinosaur track site (Lower Jurassic, eastern Free State, South Africa): a report on footprint preservation and palaeoenvironment
Source: PeerJ. 2016 Aug 23;4:e2285. doi: 10.7717/peerj.2285 (PMC5012264; doi:10.7717/peerj.2285)
Supplement: Table S1 — Additional anatomical measurements of Mafube tracks relating to the size differential between morphotypes A and B at the Mafube dinosaur track site. See Fig. 2B for how the interdigit (i.e., divarication) angle, length (t), width (w), length of rear of phalangeal part of foot (r) have been measured in this study (cf. Olsen, Smith & McDonald, 1998). All distance measurements are in centimetres; angles are in degrees. N/A: Measurements could not be determined due to e.g., absence of digit impressions; absence of heel impression; presence of the natural cast obscuring the footprint. [file peerj-04-2285-s001.pdf]

Table S1. Additional anatomical measurements of Mafube tracks relating to the size differential between morphotypes A and B at the Mafube dinosaur track site. See Figure 2B for how the interdigit (i.e., divarication) angle, length (t), width (w), length of rear of phalangeal part of foot (r) have been measured in this study (cf. Olsen et al. 1998). All distance measurements are in centimetres; angles are in degrees. N/A: Measurements could not be determined due to e.g., absence of digit impressions; absence of heel impression; presence of the natural cast obscuring the footprint.

Track #	Length	Width	Ratio length:width	r	Interdigit angle II^IV	Hypex shape II^III	Interdigit angle II^III	Hypex shape III^IV	Interdigit angle III^IV
4	41	31	1.3	23	46	N/A	23	N/A	23
6	33	27	1.2	23	57	V-shaped	26	U-shaped	31
9	26	23	1.1	18	46	N/A	21	N/A	25
11	20	21	1.0	19	54	V-shaped	31	V-shaped	23
13	N/A	15	N/A	8	49	V-shaped	21	V-shaped	28
19	28	22	1.3	20	54	V-shaped	25	V-shaped	29
21	25	20	1.3	16	52	V-shaped	24	V-shaped	28
24	21	17	1.2	10	78	N/A	33	N/A	45
25	23	18	1.3	13	N/A	N/A	N/A	N/A	N/A
26	25	17	1.5	20	49	V-shaped	22	V-shaped	27
30	32	N/A	N/A	N/A	N/A	N/A	N/A	N/A	N/A
32	30	26	1.2	N/A	N/A	N/A	N/A	N/A	N/A
37	21	15	1.4	15	42	N/A	19	N/A	23
38	29	15	1.9	14	58	N/A	30	N/A	28
39	24	17	1.4	21	N/A	N/A	N/A	N/A	N/A
41	20	16	1.3	16	N/A	V-shaped	N/A	N/A	N/A
47	13	13	1.0	11	N/A	N/A	N/A	N/A	N/A
51	N/A	18	N/A	N/A	N/A	V-shaped	N/A	V-shaped	N/A
